# Breathing pattern and pulmonary gas exchange in elderly patients with and without left ventricular dysfunction—modification with exercise-based cardiac rehabilitation and prognostic value

**DOI:** 10.3389/fcvm.2023.1219589

**Published:** 2023-09-01

**Authors:** Prisca Eser, Thimo Marcin, Eva Prescott, Leonie F. Prins, Evelien Kolkman, Wendy Bruins, Astrid E. van der Velde, Carlos Peña Gil, Marie-Christine Iliou, Diego Ardissino, Uwe Zeymer, Esther P. Meindersma, Arnoud W. J. Van’t Hof, Ed P. de Kluiver, Matthias Wilhelm

**Affiliations:** ^1^ Department of Cardiology, Inselspital, Bern University Hospital, University of Bern, Bern, Switzerland; ^2^ Department of Cardiology, Bispebjerg Frederiksberg University Hospital, Copenhagen, Denmark; ^3^ Diagram B.V., Zwolle, Netherlands; ^4^ Isala Heart Centre, Zwolle, Netherlands; ^5^ Department of Cardiology, Hospital Clínico Universitario de Santiago, University of Santiago de Compostela, Santiago de Compostela, Spain; ^6^ Department of Cardiac Rehabilitation, Assistance Publique Hopitaux de Paris, Paris, France; ^7^ Department of Cardiology, Parma University Hospital, Parma, Italy; ^8^ Klinikum Ludwigshafen and Institut für Herzinfarktforschung Ludwigshafen, Ludwigshafen, Germany; ^9^ Department of Cardiology, Radboud University, Nijmegen, Netherlands; ^10^ Department of Cardiology, Maastricht University Medical Center, Maastricht, Netherlands; ^11^ Department of Cardiology, Zuyderland Medical Center, Heerlen, Netherlands

**Keywords:** coronary artery disease, respiration, exercise training, breathing frequency, cardiopulmonary exercise testing (CPET), heart failure

## Abstract

**Background:**

Inefficient ventilation is an established prognostic marker in patients with heart failure. It is not known whether inefficient ventilation is also linked to poor prognosis in patients with left ventricular dysfunction (LVD) but without overt heart failure.

**Objectives:**

To investigate whether inefficient ventilation in elderly patients with LVD is more common than in patients without LVD, whether it improves with exercise-based cardiac rehabilitation (exCR), and whether it is associated with major adverse cardiovascular events (MACE).

**Methods:**

In this large multicentre observational longitudinal study, patients aged ≥65 years with acute or chronic coronary syndromes (ACS, CCS) without cardiac surgery who participated in a study on the effectiveness of exCR in seven European countries were included. Cardiopulmonary exercise testing (CPET) was performed before, at the termination of exCR, and at 12 months follow-up. Ventilation (VE), breathing frequency (BF), tidal volume (VT), and end-expiratory carbon dioxide pressure (P_ET_CO_2_) were measured at rest, at the first ventilatory threshold, and at peak exercise. Ventilatory parameters were compared between patients with and without LVD (based on cardio-echography) and related to MACE at 12 month follow-up.

**Results:**

In 818 patients, age was 72.5 ± 5.4 years, 21.9% were women, 79.8% had ACS, and 151 (18%) had LVD. Compared to noLVD, in LVD resting VE was increased by 8%, resting BF by 6%, peak VE_,_ peak VT, and peak P_ET_CO_2_ reduced by 6%, 8%, and 5%, respectively, and VE/VCO_2_ slope increased by 11%. From before to after exCR, resting VE decreased and peak P_ET_CO_2_ increased significantly more in patients with compared to without LVD. In LVD, higher resting BF, higher nadir VE/VCO_2_, and lower peak P_ET_CO_2_ at baseline were associated with MACE.

**Conclusions:**

Similarly to patients with HF, in elderly patients with ischemic LVD, inefficient resting and exercise ventilation was associated with worse outcomes, and ExCR alleviated abnormal breathing patterns and gas exchange parameters.

## Introduction

Ischemic heart disease is the most prevalent risk factor for left ventricular dysfunction (LVD) and chronic heart failure (HF), both for women and men (1). Impaired pulmonary gas exchange, quantified as an increased ventilation (VE) to carbon dioxide exhalation (VCO_2_) slope during exercise, and low end-tidal pressure of carbon dioxide (P_ET_CO_2_) are landmarks of heart failure patients (2, 3). These parameters received attention when several studies found higher VE/VCO_2_ slopes and lower P_ET_CO_2_ to be associated with poorer prognosis (4–6). The components of the VE/VCO_2_ slope are the arterial CO_2_ partial pressure (P_a_CO_2_), which in CHF is affected mainly by ventilation, and the pulmonary dead space to tidal volume ration (VD/VT) mainly by pulmonary perfusion abnormalities (3, 7). Exercise hyperventilation is a hallmark of a failing heart (2). Several mechanisms have been proposed as mediators of this excessive ventilatory response, including: (1) alveolar ventilation-perfusion mismatching, (2) increased humoral stimuli (e.g., lactate and H^+^) due to skeletal muscle hypoperfusion and deconditioning, (3) juxta-capillary receptor stimulation consequent to pulmonary vascular congestion and/or hypertension, (4) augmented central and peripheral chemosensitivity, and (5) an inordinately high degree of afferent neural traffic originating from within the locomotor muscles (i.e., the ergoreflex or “skeletal muscle” hypothesis). Hyperventilation is well known to stimulate sympathetic nervous activity (8). Chronic sympathetic nervous hyperactivity, in turn, may decrease the aerobic capacity of skeletal muscles by reducing capillarisation (9) and red blood cell flux (10), which lead to a shift in muscle fibre type towards a lower content on type I fibres (11). The ensuing anaerobic muscle metabolism leads to increased muscle fatiguability (12) and acidosis already at low levels of exercise, which trigger exaggerated responses in ventilation (13). While exaggerated ventilation has been found to be a strong predictor of mortality and adverse cardiac events in patients with established heart failure, this has not been assessed in patients with LVD or with ischemic heart disease (IHD). There is a continuum from patients with IHD to LVD and HF (14), with asymptomatic LVD being twice as common as HF (15). However, inefficient ventilation has not been assessed as a prognostic marker in patients with IHD or LVD.

Comprehensive exercise-based cardiac rehabilitation (exCR) has the potential to improve cardiorespiratory fitness (CRF) and quality of life (16), cardiovascular risk factors (17), and reduce hospitalisations and cardiovascular mortality (17, 18). In HF patients with reduced ejection fraction, studies assessing the effects of exercise have focussed largely on circulatory parameters (19), peak oxygen uptake (VO_2_) and muscle strength (20). Some studies have also reported changes in ventilatory efficiency in response to exercise during exCR (21, 22). However, data in elderly patients with LVD are sparse and no data exists on how exCR influences breathing patterns at rest.

The purpose of the present study in elderly patients with and without ischemic LVD was to (1) describe breathing patterns and pulmonary gas exchange parameters at rest and during exercise; (2) assess the association of exCR with a change of these parameters; and (3) assess the prognostic value of these parameters on major adverse cardiac events (MACE). We hypothesised that ventilation would be less efficient in patients with compared to those without LVD, and that, similarly to patients with HF, exCR would improve ventilatory efficiency, and that ventilatory inefficiency would be associated with MACE.

## Methods

The EU-CaRE observational longitudinal study was a European project focusing on the effectiveness and sustainability of exCR programs in the elderly (65 years or above). EU-CaRE was conducted from 2016 to 2019 and involved eight participating CR sites in seven countries, with five offering ambulatory CR of 6–12 weeks duration (Denmark, Italy, the Netherlands, Spain, and Switzerland) and two offering stationary CR of 3–4 weeks duration (France, Germany) (23).

### Study population, assessments, and intervention

Inclusion and exclusion criteria of the study population and outcome data, such as peak oxygen uptake, smoking, body mass index, diet, physical activity, serum lipids, psychological distress, and medication have been reported previously (24–28). Briefly, the inclusion criteria were patients ≥65 years old with coronary heart disease (including also valve surgery patients who were not considered for the present study). For the present study, only data from patients with acute or chronic coronary syndromes (ACS/CCS) with or without percutaneous coronary intervention (PCI) were included. Exclusion criteria were valve and cardiovascular surgery. Patients after open-chest surgery were excluded because they were found to have much greater recoveries in power output, peak oxygen consumption, and ventilatory efficiency than patients after PCI. Further, patients with open heart surgery had a lower percentage of MACE than patients after PCI (27). Patients were assessed at baseline before commencing exCR (T0), after completing the exCR program (T1), and at 1-year follow-up (T2). Detailed information about the exCR programs of the different centres was given elsewhere (23) and training intensity of the endurance training sessions of each centre have also been analysed previously (26). They were grouped according to left ventricular ejection fraction (LVEF) determined by the patient's most recent echocardiography into patients with LVD (LVEF < 45%), vs. those without LVD (noLVD, LVEF ≥ 45%). At the time when this study was designed, this cut-off was one of the more widely used and accepted cut-offs for systolic LVD and HF with reduced LVEF (HFrEF) (29).

The study was approved by all relevant medical ethics committees, and registered at trialregister.nl (NTR5306). The participants gave written informed consent before they were included in the study.

### Data collection of predictors and outcomes

Recorded information included demographics, index event, socioeconomic factors, medical history including co-mobidities and cardiovascular risk factors, and clinical information such as weight, blood pressure (BP), resting heart rate (HR), medication, SF36 quality of life questionnaire, and patient-reported physical activity as number of days per week with at least moderate physical activity of minimally 30 min. Details on the collected data have been provided elsewhere (24, 30, 31).

LVEF was determined by standard two-dimensional transthoracic echocardiography performed by the different hospital echocardiography systems. LV end-diastolic (LVEDV), and LV end-systolic volumes (LVESV) were calculated using the biplane method of disks summation (modified Simpson's rule) (32).

CRF and breathing parameters were assessed by cardiopulmonary exercise testing (CPET). After reporting to the laboratory, patients rested supine for 10–15 min during which a resting 12-lead electrocardiogram (ECG) was performed. Then patients stood up and performed a resting spirometry with the determination of forced vital capacity (FVC) and forced expiratory volume during the first second (FEV1). After this, patients mounted the cycle ergometers and were fitted with a facemask. Then they remained sitting quietly for 3 min during which heart rate, blood pressure, and breathing parameters were measured. As an average of the last minute of the 3-min resting period, while sitting on the ergometer, the following parameters were determined: minute ventilation (VE), breathing frequency (BF), tidal volume (VT), partial pressure of carbon dioxide (P_ET_CO_2_), and heart rate. After a 3-min warm-up at 5 Watt an individual ramp was chosen to achieve a test duration of 8–12 min until exhaustion and kept constant in follow-up tests. Data were analysed at the CPET core lab in Bern as previously described (27, 31). During the ramp, the VE/VCO_2_ slope, and the nadir of the VE/VCO_2_ ratio were determined. Oxygen consumption (VO_2_), VE, BF, VT, P_ET_CO_2_, and heart rate were determined at the first ventilatory threshold as previously described (27). Peak values of the same parameters were determined as the highest value of a 30 s moving average. Ventilatory parameters were excluded when the respiratory quotient was below 0.7 at resting and below 0.8 at peak exercise due to the suspected presence of mask leakage.

MACE was defined as the combined incidence of all-cause mortality, ACS, cardiac-related emergency visit, hospitalisation for cardiac reasons, near sudden cardiac death, and cardiac intervention. MACE was recorded by monthly telephone calls and assessed individually by an independent Clinical Event Committee (27).

### Statistical analysis

All statistics were performed with R (Version 4.1.0, R Core Team, 2021). Changes in ventilatory and circulatory parameters were calculated between T0 and T1. Mixed linear models were performed for ventilatory parameters VE, BF, VT, and P_ET_CO_2_ at rest and peak exercise, as well as VE/VCO_2_ slope and peak VO_2_ relative to body weight and tested for interaction effects between time points T0, T1, and T2 and group (with and without LVD). All models were adjusted for age, sex, and body mass index (BMI), and patients nested within centres were entered as random factors (intercepts). We chose mixed linear models because they can handle the presence of missing data and allow assessment of longitudinal data and adjustment for confounders, such as centres, age, sex, and BMI.

The association of ventilatory parameters at rest and during exercise with MACE was also assessed with mixed linear models for patients with and without LVD and with and without MACE. For all models, the alpha was set at 0.05.

## Results

**Figure 1:**
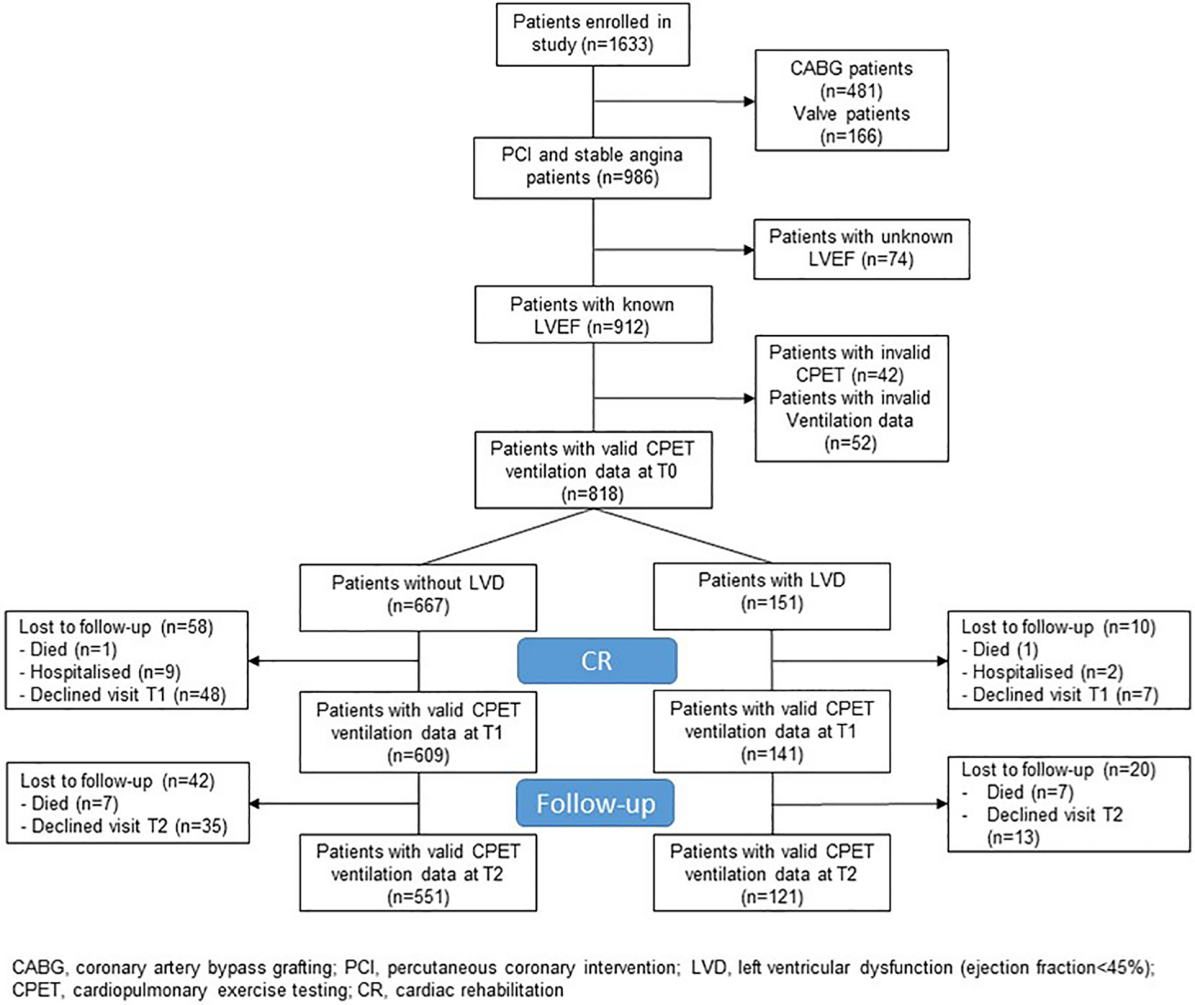
Study flow.

Of 1,633 enrolled EU-CaRE patients, 986 patients had an ACS or CCS with percutaneous coronary intervention or no revascularisation. Of these, 79.8% had an ACS and 90.2% a PCI as an indication for exCR. LVEF was known in 912 patients. Of these, valid CPET at T0 was available in 867 patients of whom 707 had no LVD and 166 had LVD. Amongst these, data on breathing patterns were available from at least one time point in 151 with and 667 patients without LVD (Figure 1). The baseline data of the two patient groups are shown in Table 1. Patients with LVD were older, were less often women, and had lower BMI and FVC relative to body surface area. An equal prevalence of 86% of patients with New York Heart Association (NYHA) functional class I existed in patients with and without LVD, and NYHA III class was present in 2% and 3% of patients with and without LVD, respectively. Patients with LVD had significantly lower physical component scores of the SF36 indicating poorer health status. Patients with LVD had a higher percentage of pre-existing chronic heart failure and anaemia, and a higher prescription rate of beta-blockers and ACE inhibitors or ARBs.

**Table 1:** Baseline characteristics of patients without vs. with LVD. Parameters are indicated as mean ± SD, *n* (%), or median [1st, 3rd quartile]. *P*-values are derived from unpaired T-tests, Wilcoxon tests, Chi-square or Fisher’s exact test, as appropriate.

Group (*n*)	No LVD (667)	LVD (151)	*p*-value
Age [years]	72.3 ± 5.4	73.3 ± 5.4	0.0340
Female sex	156 (23.4)	21 (13.9)	0.0145
BMI [kg/m^2^]	27.7 ± 4.0^c^	26.8 ± 3.9^a^	0.0036
Forced vital capacity [L]	3.31 ± 0.84^c^	3.17 ± 0.87^a^	0.0952
Forced vital capacity/BSA [L/m^2^]	1.14 ± 0.24^c^	1.08 ± 0.24^a^	0.0067
Forced expiratory volume in the first second [L/s]	2.59 ± 0.69^c^	2.49 ± 0.64^a^	0.0705
Patient-reported outcomes			
NYHA class I/II/III	574/73/17^a^	130/18/3	0.8776
SF36 physical component score	47.5 [41.1, 52.6]^e^	44.3 [38.8, 49.4]^d^	0.0004
Days with >30 min physical activity	3 [1, 7]^b^	4 [2, 7]^a^	0.2366
Cardiac diseases			
Indication for exCR ACS	516 (77.4)	129 (85.4)	0.0373
PCI during index event	598 (89.7)	140 (92.7)	0.3215
Previous ACS	141 (21.1)^a^	42 (27.8)	0.1028
Chronic heart failure	3 (0.4)	10 (6.6)	0.0000
Atrial fibrillation	33 (4.9)	12 (7.9)	0.2069
CV risk factors			
Smoking (active/former/never)	69/111/485^a^	14/25/112	0.9163
Hypertension	438 (65.7)	100 (66.2)	0.9901
Hypercholesterolemia	453 (67.9)	94 (62.3)	0.1974
Diabetes mellitus	152 (22.8	43 (28.5)	0.1751
Family history of CVD	162 (24.3)	35 (23.2)	0.9056
Comorbidities			
Nephropathy	47 (7.0)	17 (11.3)	0.1102
Peripheral arterial disease	44 (6.6)	12 (7.9)	0.6630
Chronic obstructive pulmonary disease	41 (6.1)	14 (9.3)	0.2199
Obstructive sleep apnea	22 (3.3)	2 (1.3)	0.3076
Anaemia	116 (29.0)^f^	42 (49.4)^g^	0.0179
Medication			
Beta-blockers	510 (76.5)	140 (92.7)	0.0001
Statins	637 (95.5)	144 (95.4)	0.9999
ACE inhibitors or ARBs	501 (75.1)	130 (86.1)	0.0056
Diuretics	155 (23.2%)	83 (55.0%)	0.0000

LVD, left ventricular dysfunction; BMI, body mass index; NYHA, New York heart failure association; SF36, short-form health survey; CV, cardiovascular; ACS, acute coronary syndrome; CVD, cardiovascular disease; ACE, angiotensin-converting enzyme; ARB, angiotensin receptor blockers; BSA, body surface area.

^a^ Missing data ≥1 and ≤5.

^b^ Missing data ≥6 and ≤10.

^c^ Missing data ≥11 and ≤15.

^d^ Missing data = 17.

^e^ Missing data = 40.

^f^ Missing data = 151.

^g^ Missing data = 29.

With regard to ventilatory parameters, two centres did not record data on BF and VT, therefore, these parameters were missing in 5 and 51 patients with and without LVD, respectively. Due to an insufficient ventilation monitoring duration (≤1 min) during the 3-min resting phase, VE and P_ET_CO_2_ at rest were missing in 9 and 68 patients with and without LV dysfunction at T0, respectively, in 6 and 82 at T1, respectively, and in 4 and 46 at T2, respectively. The first ventilatory threshold could not be determined in 38 and 130 patients with and without LV dysfunction, respectively, at T0, in 20 and 71 at T1, respectively, and in 16 and 61 at T2, respectively. The largest relative adjusted (for sex, age, and BMI) differences of the LVD group compared to the noLVD group at baseline were found for VO_2_ peak (−12.6%), VE/VCO_2_ slope (11.0%), nadir VE/VCO_2_ (8.8%), and resting VE (8.1%) (Table 2).

**Table 2:** Ventilatory and circulatory parameters at rest, during ramp exercise (at first ventilatory threshold), and at peak exercise for patients without vs. patients with LVD before the start of exCR as well as change until completion of CR. Shown are medians and first and third quartiles in round brackets. The relative difference between groups is derived from group effect from adjusted models and is relative to values from groups without LVD. *P*-values are derived from mixed linear models adjusted for age, sex, and body mass index and with patients nested within centres as random intercepts.

Group (*n*)	Patients without LVD (667)	Patients with LVD (151)	Relative difference [%]	*p*-value for group main effect	Change with CR in patients without LVD (609)	Change with CR in patients with LVD (141)	*p*-value for group × time interaction
Resting							
Ventilation [L/min]	12.4 (10.2, 14.9)	13.1 (11.6, 16.2)	**8**.**1**	**0**.**0019**	0.12 (−1.63, 1.83)	−0.93 (−2.96, 1.13)	**0**.**0018**
Breathing frequency [min^−1^]	17.2 (14.5, 20.5)	18.0 (14.9, 21.3)	**5**.**8**	**0**.**0126**	0.03 (−2.17, 2.13)	−0.72 (−3.29, 2.03)	0.1346
Tidal volume [ml/breath]	736 (570, 895)	739 (604, 905)	0.0	0.9543	1.30 (−133, 141)	−15.1 (−171, 106)	0.3896
P_ET_CO_2_ [mmHg]	29.2 (26.7, 32.2)	27.4 (24.6, 30.3)	−2.3	0.0537	0.14 (−1.68, 1.99)	0.11 (−1.68, 2.22)	0.5562
Heart rate [bpm]	63 (58, 70)	65 (60, 74)	**3**.**8**	**0**.**0245**	−2 (−8, 4)	−3 (−9, 3)	0.9148
At first ventilatory threshold							
VO_2_ [ml/kg/min]	10.9 (9.25, 12.6)	9.7 (8.6, 11.7)	−**7**.**5**	**0**.**0024**	1.13 (−0.26, 2.83)	0.92 (−0.50, 2.33)	0.6841
Ventilation [ml/min]	341 (288, 403)	333 (280, 411)	−1.5	0.2477	37 (−13, 92)	26 (−29, 80)	0.6843
Breathing frequency [min^−1^]	20.5 (18.3, 24.4)	21.4 (18.3, 24.4)	**5**.**1**	**0**.**0441**	0.57 (−1.37, 2.59)	−0.03 (−2.09, 2.50)	0.3010
Tidal volume [ml/breath]	1,338 (1,126, 1,627)	1,239 (998, 1,523)	−**6**.**7**	**0**.**0120**	110 (−48, 268)	65 (−111, 282)}	0.9715
P_ET_CO2 [mmHg]	34.4 (31.7, 37.3)	32.2 (28.4, 35.1)	−**5**.**1**	**0**.**0000**	0.35 (−1.42, 1.88)	1.32 (−0.55, 3.25)	0.0620
During ramp exercise							
VE/VCO_2_ slope	32.1 (28.5, 37.2)	36.6 (31.0, 43.1)	**11**.**0**	**0**.**0000**	0.09 (−3.28, 2.88)	−1.45 (−4.45, 1.56)	0.3327
Nadir VE/VCO_2_	33.7 (30.8, 37.7)	36.7 (33.5, 42.8)	**8**.**8**	**0**.**0000**	−0.09 (−1.96, 1.51)	−1.21 (−3.42, 1.00)	**0**.**0407**
At peak exercise							
VO_2_ [ml/kg/min]	17.1 (14.1, 20.1)	15.4 (12.8, 18.0)	−**12**.**6**	**0**.**0000**	1.29 (−0.06, 3.08)	1.39 (0.05, 2.87)	0.6422
Ventilation [L/min]	54.2 (42.7, 68.5	53.7 (41.9, 65.0)	−**5**.**5**	**0**.**0175**	5.13 (−0.54, 11.58)	6.22 (−2.30, 12.15)	0.6222
Breathing frequency [min^−1^]	32.5 (28.3, 36.8)	32.9 (28.7, 37.4)	1.0	0.6166	1.75 (−1.18, 4.86)	1.96 (−0.49, 4.72)	0.5397
Tidal volume [L/min]	1.90 (1.54, 2.33)	1.76 (1.43, 2.18)	−**7**.**9**	**0**.**0005**	0.08 (−0.07, 0.19)	0.08 (−0.09, 0.21)	0.3680
Maximal P_ET_CO_2_ [mmHg]	35.7 (32.7, 38.6)	33.6 (29.7, 36.6)	−**5**.**4**	**0**.**0000**	0.18 (−1.24, 1.76)	0.97 (−0.50, 2.81)	**0**.**0055**
RER (VCO_2_/VO_2_)	1.08 (1.01, 1.14	1.04 (0.97, 1.12)	−**1**.**8**	**0**.**0266**	0.01 (−0.04, 0.07)	0.04 (−0.02, 0.10)	**0**.**0284**
Heart rate [bpm]	114 (103, 130)	109 (97, 125)	−1.7	0.2608	2.8 (−3.9, 9.8)	5.7 (−2.5, 14.0)	0.1691

LVD, left ventricular dysfunction; PetCO2, end-tidal partial pressure of CO2; VO2, oxygen uptake, VE, ventilation; VCO2, carbon dioxide production.

*P*-values <0.05 and respective relative differences are marked in bold.

Regarding the changes in ventilatory parameters from before to after CR, patients with LVD had significantly greater reductions in resting VE (Table 2). They also had greater reductions in nadir VE/VCO_2_, and a greater increase in P_ET_CO_2_ and RER at peak exercise.

**Figure 2:**
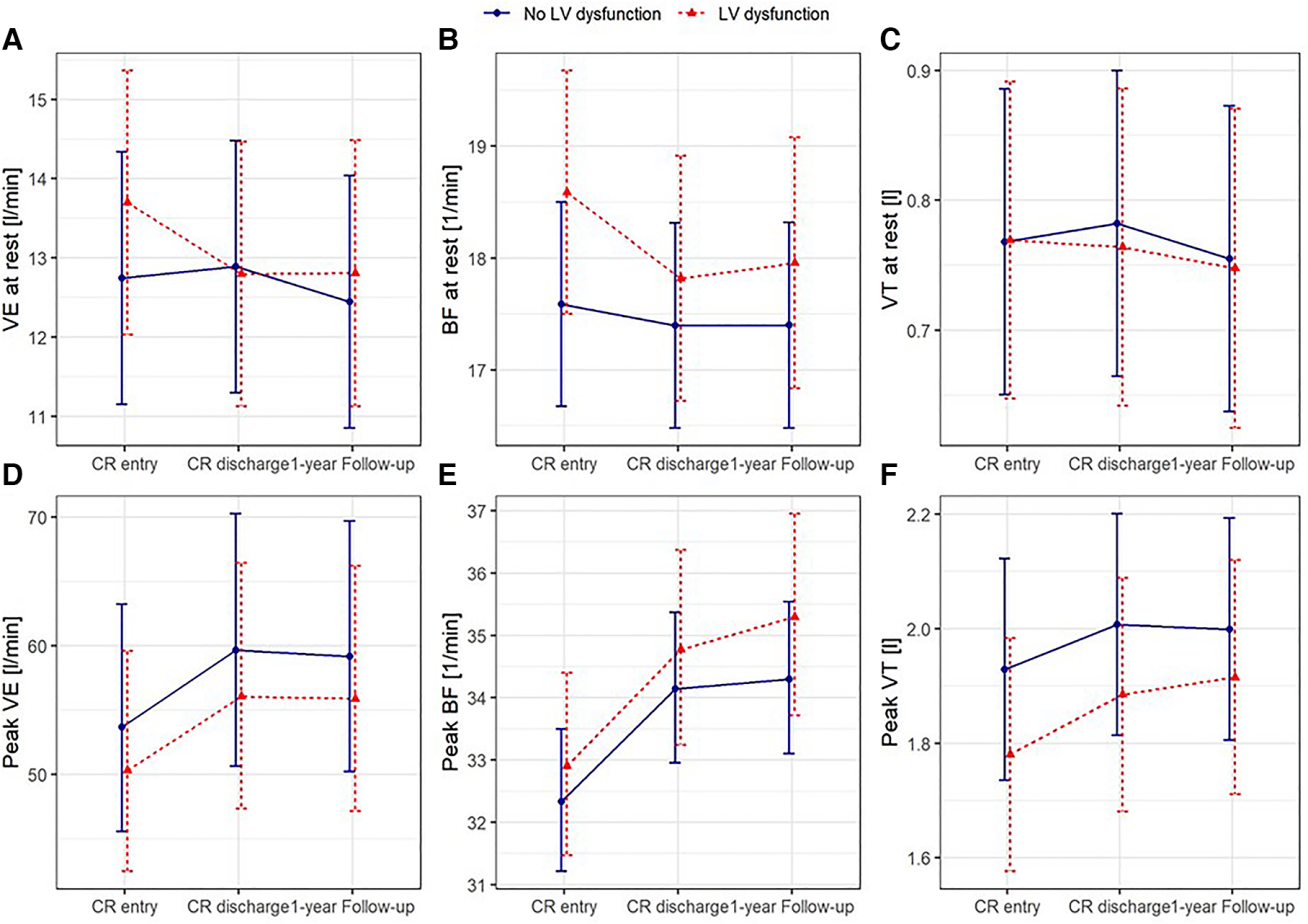
Predicted means with 95% confidence intervals based on the mixed linear models for ventilation (**A**), breathing frequency (**B**), and tidal volume (**C**) at rest and at peak exercise (**D–F**) adjusted for age, sex, and body mass index, with patients nested within centres as random intercepts.

**Figure 3:**
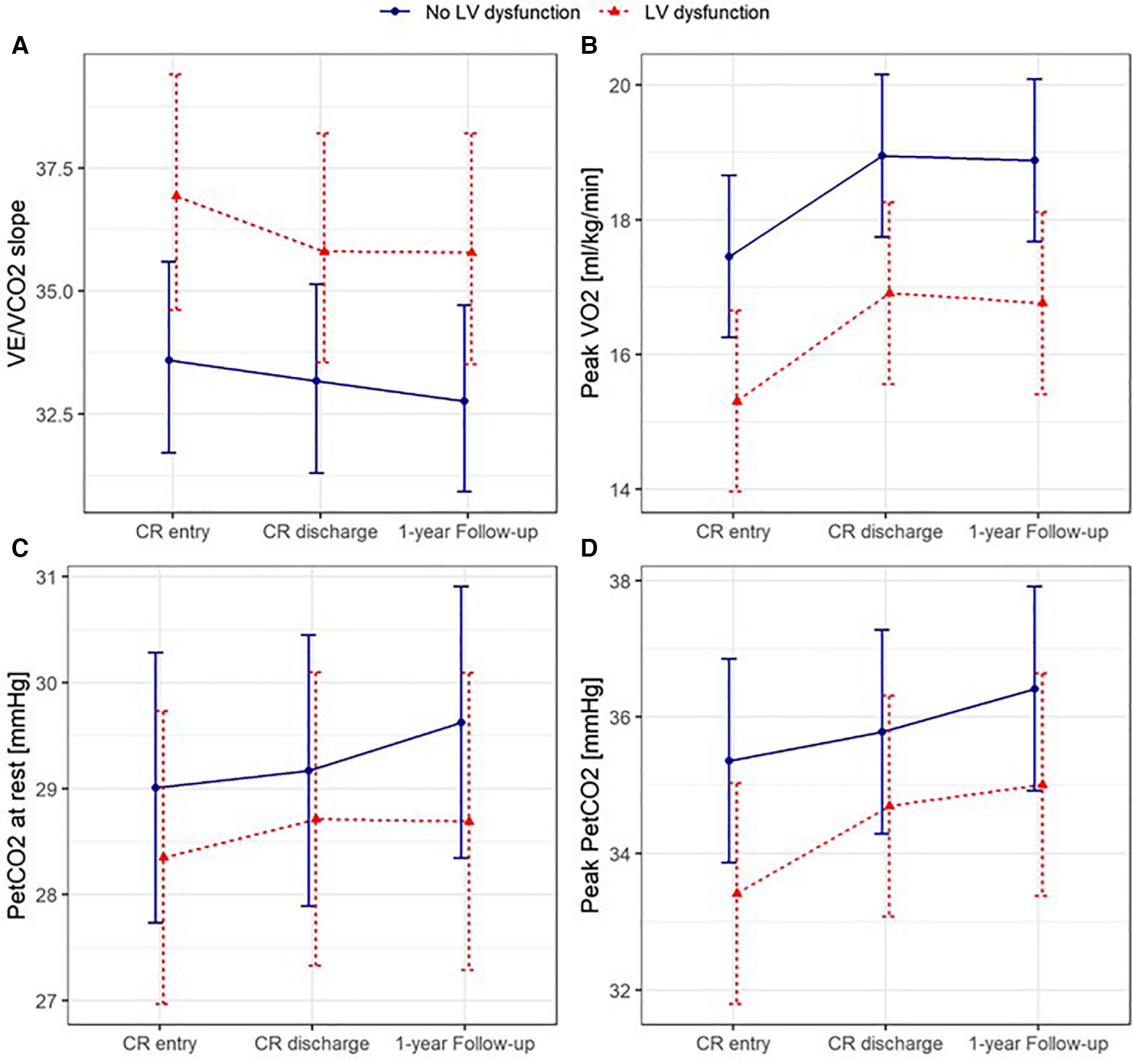
Predicted means with 95% confidence intervals based on the mixed linear models for VE/VCO_2_ slope (**A**), peak VO_2_ (**B**), and end-tidal carbon dioxide partial pressure at rest (**C**) and at peak exercise (**D**) adjusted for age, sex, and body mass index, with patients nested within centres as random intercepts.

Results from the mixed linear models including data also for the 1-year follow-up measurement as well as confounding factors age, sex, and BMI showed a significantly greater reduction of VE at rest in the group of patients with LVD not only from before to after exCR but also to 1-year follow-up (Figure 2, Supplementary Table S1). BF at rest was higher in patients with LVD at all time points (Figure 2, Supplementary Table S1), and resting P_ET_CO_2_ increased significantly at 1-year follow-up in both groups (Figure 3, Supplementary Table S1). At peak exercise, VE was reduced in the patients with LVD due to a significantly lower VT in the patients with LVD but increased with exCR and to 1-year follow-up in both groups (Figure 2, Supplementary Table S2). Resting P_ET_CO_2_ was lower by trend (*p* = 0.0537) in patients with LVD and significantly lower at peak exercise at all time points (Figure 3, Supplementary Tables S1 and S2). Accordingly, VE/VCO_2_ slope was increased in patients with LVD at all time points (Figure 3, Supplementary Table S3). VO_2_ relative to body weight was greatly reduced in patients with LVD at peak exercise (Figure 3, Supplementary Table S3) at all time points. Peak VO_2_ increased in both groups from before to after exCR and VE/VCO_2_ slope decreased over time. The physical component score was consistently lower by 3 points in patients with LVD but significant consistent improvements in SF36 PCS between time points were comparable in both groups with nearly 3 points between T0 and T2 (Supplementary Table S3).

Approximately 20% of patients with and without LVD had a MACE within 1-year follow-up with no difference between groups (*p*-value for Chi-square test 0.069). Nadir of VE/VCO_2_ significantly discriminated patients with and without MACE in patients with and without LVD, while VE/VCO_2_ slope and peak VO_2_ discriminated for MACE only in patients without LVD, and peak P_ET_CO_2_ and resting BF discriminated for MACE only in patients with LVD (Table 3).

**Table 3:** Association of MACE with CPET parameter. Shown are estimates and 95% CI for MACE from the mixed linear models with the respective CPET parameter as the dependent variable and covariates age and sex, and with site as random factor. Models were performed for patients with and without LVD and for the total population to test for group interaction.

	LVD (*n* = 151, 25 with MACE)		No LVD (*n* = 667, 104 with MACE)		Interaction MACE × LVD
	Beta	95%CI	Beta	95%CI	*p*-value
VO_2_ peak [ml/kg/min]	−1.150	−2.842, 0.520	−1.249	−2.136, −0.354	0.7608
VE/VCO_2_ slope	3.448	−0.092, 7.046	2.244	0.757, 3.739	0.3159
Nadir VE/VCO_2_	2.843	0.071, 5.615	1.189	0.091, 2.294	0.1022
P_ET_CO_2_ resting [mmHg]	−0.951	−2.573, 0.653	−0.250	−1.093, 0.593	0.3158
P_ET_CO_2_ peak [mmHg]	−1.880	−3.759, −0.019	−0.404	−1.328, 0.515	0.0860
VE resting [L/min]	0.882	−0.853, 2.632	−0.795	−1.613, 0.027	0.0742
VE peak [L/min]	2.644	−3.855, 9.105	−2.302	−5.390, 0.803	0.3411
BF resting [min^−1^]	2.985	0.639, 5.418	−0.688	−2.097, 0.714	0.0086
BF peak [min^−1^]	2.928	−0.265, 6.122	1.200	−0.506, 2.888	0.5638
VT resting [L]	−0.055	−0.165, 0.054	−0.018	−0.073, 0.038	0.4746
VT peak [L]	−0.155	−0.334, 0.021	−0.075	−0.173, 0.023	0.2658

LVD, left ventricular dysfunction; MACE, major adverse cardiac event: VO_2_, oxygen uptake; VE/VCO_2_, ventilation/carbon dioxide production; P_ET_CO_2_, endtidal carbon dioxide partial pressure; BF, breathing frequency; VT, tidal volume.

## Discussion

This study provides four clinically relevant and novel aspects in a representative European cohort of elderly patients with coronary artery disease undergoing exCR: (1) Nearly one-fifth of patients had asymptomatic LVD at the start of exCR; (2) LVD was associated with elevated ventilation at rest and impaired pulmonary gas exchange during exercise; (3) exCR was associated with an improvement of ventilation at rest and gas exchange parameters during exercise, but ventilatory efficiency and P_ET_CO2 remained significantly reduced in patients with LVD compared to patients without LVD in the 1-year follow-up; and (4) BF at rest, ventilatory efficiency and P_ET_CO_2_ during exercise were associated with worse outcome in patients with LVD.

This is the first large study demonstrating increased resting ventilation and impaired pulmonary gas exchange during exercise in patients with LVD without major signs and symptoms of congestive heart failure. NYHA functional class did not differ between patients with and without LVD and 86% of patients in both groups were in NYHA functional class I. Furthermore, self-reported physical activity did not differ between the groups. Although the median physical component score of the SF36 questionnaire was 3 points lower in patients with LVD compared to patients without LVD, the values of 44.3 at T0 and 47.5 at T2 were clearly above the median of 35.3 found in patients with chronic heart failure (33).

We suggest that signs of an exaggerated ventilatory drive are already apparent in patients with LVD at rest (higher resting VE and BF), during exercise (increased VE/VCO_2_ slope and nadir VE/VCO_2_), and at peak exercise (lower VO_2_, VT, and P_ET_CO_2_). Reductions of the exaggerated ventilatory drive seen with exCR were similarly beneficial in patients with and without LVD. The decrease in nadir VE/VCO_2_ was significantly greater in patients with LVD than in those without LVD, with a similar magnitude of the difference between changes in VE/VCO_2_ slope, albeit not reaching statistical significance. This is in accordance with some previous studies that found ventilatory efficiency to improve most in patients with the most severe ventilatory inefficiency at baseline. For example, in a study with 131 patients after acute myocardial infarction without HF participating in exCR, VE/VCO_2_ slope improved most in patients with VE/VCO_2_ slope > 32 (34), which was also confirmed by a study comparing the effect of aerobic training to a combination of aerobic and strength training (35). Similarly, a study in patients with HFrEF by Servantes et al. found large decreases in VE/VCO_2_ slopes with mean pre-exercise values of 36 (36). In part, a larger decrease in patients with higher starting values may be due to a phenomenon referred to as “regression to the mean” (37). In a study including 123 CAD patients, patients in the group with peak VO_2 _< 17.5 ml/kg/min also had the highest nadir VE/VCO_2_ (38). This group also had the highest improvement in peak VO_2_ and nadir VE/VCO_2_ after exCR. Likewise, in 37 CAD patients completing exCR, P_ET_CO_2_ at the first and second ventilatory threshold and peak exercise were improved after the programme. On the contrary, Fu et al. did not demonstrate an improvement of the VE/VCO_2_ slope with exercise training in patients with HFrEF and a VE/VCO_2_ slope of 35 at baseline (21).

At peak exercise, peak ventilation was 3 L/min (6%) lower in patients with compared to patients without LVD, while peak VO_2_ was reduced by 2.2 ml/kg/min (12%). The disproportionate reduction in gas exchange was likely to be due to the significantly smaller peak VT (by 150 ml or 8%) in patients with LVD at an insignificantly increased peak BF, resulting in a larger anatomical dead space. This is in line with observation in patients with HFrEF (39), and the emerging evidence that HF patients adopt a “rapid shallow breathing pattern” to avoid the adverse effects of large intrathoracic pressure swings on cardiac pre- and afterload (2). Importantly, our study extends the evidence on abnormal breathing patterns and hyperventilation in patients with HFrEF to patients with LVD. The significantly higher resting HR in patients with LVD compared to patients without LVD is compatible with sympathetic hyperactivity (2, 40). This finding suggests that several proposed mechanisms for exaggerated ventilation in HFrEF (e.g., augmented peripheral and central chemosensitivity and an inordinately high degree of afferent neural traffic originating from within the locomotor muscles) may already be present in patients with asymptomatic LVD starting the vicious cycle that may progress LVD to HFrEF (2). Interestingly, the models corrected for age, sex, and BMI showed a significant positive relationship of BMI with P_ET_CO_2_ at rest and at AT, and a significant inverse relationship with nadir VE/VCO_2_. This raises the question of whether the obesity paradox not only applies to patients with HF but already to elderly patients with (and without) LVD (41).

The observed normalisation of the excessive ventilation of patients with LVD after exCR may partly be ascribed to the beneficial effects of exercise training on ergoreflex sensitivity, and/or chemoreceptor activation (40). Importantly, guideline-directed medical and device therapies for HFrEF improve chemo- and baroreceptor function insufficiently, highlighting the importance of exercise in this population (42).

While the VE/VCO_2_ slope is an established prognostic parameter in patients with HFrEF, only a few studies have reported the physiologically inversely related parameter P_ET_CO_2_ at rest and at peak exercise (43, 44). Matsumoto and colleagues have found the severity of HF in 112 patients with cardiac disease to be negatively associated with P_ET_CO_2_ at rest and at peak exercise (43). They found P_ET_CO_2_ at peak exercise to be negatively associated with VE/VCO_2_ slope and VD/VT, but not with P_a_CO_2_. Arena and colleagues found a resting P_ET_CO_2_ of <33 mmHg to discriminate significantly for cardiac events at 1-year follow-up and to significantly add discriminative power to VE/VCO_2_ slope (44). Similarly, Schäper and colleagues found resting P_ET_CO_2 _≤ 31 mmHg to be associated with mortality (45). Our study demonstrated consistent findings on the prognostic value of pulmonary gas exchange parameters in patients with LVD. The nadir VE/VCO_2_ and peak P_ET_CO_2_ were associated with MACE. Moreover, as a novel finding, BF at rest was associated with MACE. The discriminative value of resting ventilation parameters for MACE deserves further investigation as both resting P_ET_CO_2_ and BF have been found to be predictive for cardiovascular complications also in lung resection patients (46). Interestingly, NYHA class was not associated with MACE.

The important role of low P_ET_CO_2_ in patients with HFrEF and periodic breathing has been pointed out by a study by Apostolo and colleagues who restored a normal breathing pattern with inhalation of 2% CO_2_ in 95% of their study patients (47). Likewise, inhalation of CO_2_ has been shown to stabilise breathing in patients with periodic breathing and Cheyne-Stokes respiration during sleep (48–50). Breathing instabilities are recognised markers for poor prognosis in patients with HFrEF and highlight the central role of breathing patterns in the progression of HF (51). Nevertheless, few therapies have been developed to address breathing patterns. A few small studies with encouraging results have employed slow breathing training in patients with heart failure (52, 53).

Strengths of this study are the large sample size of a representative European cohort of elderly patients with coronary artery disease undergoing exCR, and the longitudinal as well as comprehensive assessment of ventilatory parameters at rest, during ramp exercise, and at peak exercise. Furthermore, this is the first documentation of improved breathing patterns and pulmonary gas exchange both at rest and with exercise after completion of exCR in both patients with and without LVD.

One limitation of this study are its observational design which does not allow the identification of cause-effect relationships. This explorative study is also limited by multiple testing. Therefore, confidence intervals and *p*-values have to be interpreted accordingly. Unfortunately, we did not have data on beta-blocker classes. Previous studies showed that carvedilol, an unselective ß1- and ß2-blocker, was associated with a lower VE/VCO_2_ slope and nadir VE/VCO_2_ (54, 55). However, the standard treatment of ACS and CCS includes cardioselective beta-blockers like metoprolol. We can therefore only speculate that only a small proportion of the 93% of our patients have received an unselective beta-blocker. Further, ACE inhibitors have been shown to improve gas transfer and ventilation perfusion matching (56). Nevertheless, the higher percentage of ACE in patients with LVD could not completely offset their less efficient ventilation.

Logistic regressions for the identification of CPET variables significantly improving MACE prediction were performed for the whole EU-CaRE cohort (including patients with open heart surgery and valve replacement) recently (27). Due to the small sample size of our subgroup of patients with LVD, we did not perform logistic regression models in the present study.

## Conclusion

Patients with LVD had an exaggerated BF at rest and impaired pulmonary gas exchange during exercise. Abnormal breathing patterns may be an early and clinically relevant sign of LVD and linked to increased ergoreflex sensitivity and/or abnormal chemosensitivity. ExCR may contribute to improvements in breathing patterns and pulmonary gas exchange in this population. However, interventions aimed at specifically improving altered breathing patterns at rest and during exercise may have additive value and should be investigated.

## Data Availability

The datasets presented in this article are not readily available because some centres have not requested permission to make data publicly accessible. Requests to access the datasets should be directed to arnoud.vant.hof@mumc.nl.
