# Supplementary material for: Breathing pattern and pulmonary gas exchange in elderly patients with and without left ventricular dysfunction—modification with exercise-based cardiac rehabilitation and prognostic value
Source: Front Cardiovasc Med. 2023 Sep 1;10:1219589. doi: 10.3389/fcvm.2023.1219589 (PMC10505741; doi:10.3389/fcvm.2023.1219589)
Supplement: Supplementary file 1 [file Datasheet1.docx]

Supplement Table 1: Mixed linear models for resting breathing parameters VE, BF, VT and P_ET_CO_2_ and fixed effects time point (visit) and group (with and without LVD), adjusted for age, sex and BMI. Patients nested within centres were entered as random factors (intercepts only). Reference categories are Visit T0, no LVD, and female gender.

|  | Estimate | Standard error | p-value |
| --- | --- | --- | --- |
| *Resting VE [l/min]* |  |  |  |
| Intercept | 8.56 | 1.90 | 0.0000 |
| Visit T1 | 0.10 | 0.14 | 0.4690 |
| Visit T2 | -0.21 | 0.15 | 0.1437 |
| LVD | 1.01 | 0.32 | 0.0019 |
| Age [years] | -0.01 | 0.32 | 0.7376 |
| Male gender | 2.45 | 0.25 | 0.0000 |
| BMI [kg/m2] | 0.12 | 0.03 | 0.0000 |
| Visit T1 x LVD | -1.00 | 0.32 | 0.0018 |
| Visit T2 x LVD | -0.81 | 0.34 | 0.0166 |
|  |  |  |  |
| *Resting BF [1/min]* |  |  |  |
| Intercept | 12.94 | 2.15 | 0.0000 |
| Visit T1 | -0.19 | 0.18 | 0.2778 |
| Visit T2 | -0.19 | 0.18 | 0.2976 |
| LVD | 1.00 | 0.40 | 0.0126 |
| Age [years] | 0.06 | 0.02 | 0.0090 |
| Male gender | -0.72 | 0.31 | 0.0223 |
| BMI [kg/m2] | 0.02 | 0.03 | 0.5309 |
| Visit T1 x LVD | -0.58 | 0.39 | 0.1346 |
| Visit T2 x LVD % | -0.45 | 0.41 | 0.2761 |
|  |  |  |  |
| *Resting VT [l]* |  |  |  |
| Intercept | 0.788 | 0.126 | 0.0000 |
| Visit T1 | 0.014 | 0.010 | 0.1726 |
| Visit T2 | -0.013 | 0.010 | 0.2120 |
| LVD | 0.001 | 0.022 | 0.9543 |
| Age [years] | -0.004 | 0.001 | 0.0006 |
| Male gender | 0.185 | 0.017 | 0.0000 |
| BMI [kg/m2] | 0.006 | 0.002 | 0.0008 |
| Visit T1 x LVD | -0.019 | 0.023 | 0.3896 |
| Visit T2 x LVD | -0.009 | 0.024 | 0.7122 |
|  |  |  |  |
| *Resting P_ET_CO_2_ [mmHg]* | |  |  |
| Intercept | 30.96 | 1.83 | 0.0000 |
| Visit T1 | 0.16 | 0.15 | 0.2996 |
| Visit T2 | 0.62 | 0.16 | 0.0001 |
| LVD | -0.66 | 0.34 | 0.0537 |
| Age [years] | -0.10 | 0.02 | 0.0000 |
| Male gender | -0.30 | 0.26 | 0.2399 |
| BMI [kg/m2] | 0.17 | 0.03 | 0.0000 |
| Visit T1 x LVD | 0.20 | 0.34 | 0.5562 |
| Visit T2 x LVD | -0.28 | 0.36 | 0.4459 |

VE, ventilation; T1, CR start; T2, CR end; LVD, left ventricular dysfunction; BMI, body mass index; BF, breathing frequency; VT, tidal volume; P_ET_CO_2_, endtidal carbon dioxide partial pressure

Supplement Table 2: Mixed linear models for peak breathing parameters VE, BF, VT and P_ET_CO_2_ and fixed effects time point (visit) and group (with and without LVD), adjusted for age, sex and BMI. Patients nested within centres were entered as random factors (intercepts only). Reference categories are Visit T0, no LVD, and female gender.

|  | Estimate | Standard error | p-value |
| --- | --- | --- | --- |
| *Peak VE [l/min]* |  |  |  |
| Intercept | 88.01 | 7.33 | 0.0000 |
| Visit T1 | 5.86 | 0.42 | 0.0000 |
| Visit T2 | 4.84 | 0.44 | 0.0000 |
| LVD | -2.96 | 1.24 | 0.0175 |
| Age [years] | -0.68 | 0.08 | 0.0000 |
| Male gender | 20.59 | 1.01 | 0.0000 |
| BMI [kg/m2] | 0.07 | 0.10 | 0.4683 |
| Visit T1 x LVD | -0.48 | 0.97 | 0.6222 |
| Visit T2 x LVD | 0.86 | 1.02 | 0.4007 |
|  |  |  |  |
| *Peak BF [1/min]* |  |  |  |
| Intercept | 31.61 | 3.75 | 0.0000 |
| Visit T1 | 1.65 | 0.26 | 0.0000 |
| Visit T2 | 1.75 | 0.27 | 0.0000 |
| LVD | 0.24 | 0.68 | 0.7267 |
| Age [years] | 0.01 | 0.04 | 0.8794 |
| Male gender | 0.11 | 0.55 | 0.8401 |
| BMI [kg/m2] | 0.05 | 0.06 | 0.3906 |
| Visit T1 x LVD | 0.32 | 0.59 | 0.5858 |
| Visit T2 x LVD | 0.96 | 0.62 | 0.1193 |
|  |  |  |  |
| *Peak VT [l]* |  |  |  |
| Intercept | 3.40 | 0.25 | 0.0000 |
| Visit T1 | 0.08 | 0.01 | 0.0000 |
| Visit T2 | 0.07 | 0.01 | 0.0000 |
| LVD | -0.15 | 0.04 | 0.0005 |
| Age [years] | -0.03 | 0.00 | 0.0000 |
| Male gender | 0.71 | 0.04 | 0.0000 |
| BMI [kg/m2] | -0.01 | 0.00 | 0.0597 |
| Visit T1 x LVD | 0.02 | 0.03 | 0.3680 |
| Visit T2 x LVD | 0.05 | 0.03 | 0.0551 |
|  |  |  |  |
| *Peak P_ET_CO_2_ [mmHg]* |  |  |  |
| Intercept | 41.70 | 2.29 | 0.0000 |
| Visit T1 | 0.43 | 0.14 | 0.0020 |
| Visit T2 | 1.06 | 0.14 | 0.0000 |
| LVD | -1.94 | 0.40 | 0.0000 |
| Age [years] | -0.14 | 0.03 | 0.0000 |
| Male gender | 0.52 | 0.33 | 0.1144 |
| BMI [kg/m2] | 0.12 | 0.03 | 0.0004 |
| Visit T1 x LVD | 0.86 | 0.31 | 0.0055 |
| Visit T2 x LVD | 0.54 | 0.32 | 0.0988 |

VE, ventilation; T1, CR start; T2, CR end; LVD, left ventricular dysfunction; BMI, body mass index; BF, breathing frequency; VT, tidal volume; P_ET_CO_2_, endtidal carbon dioxide partial pressure

Supplement Table 3: Mixed linear models for VE/VCO_2_ slope, peak VO_2_ and SF36 physical component score with fixed effects time point (visit) and group (with and without LVD), adjusted for age, sex and BMI. Patients nested within centres were entered as random factors (intercepts only). Reference categories are Visit T0, no LVD, and female gender.

|  | Estimate | Standard error | p-value |
| --- | --- | --- | --- |
| *VE/VCO_2_ slope* |  |  |  |
| Intercept | 17.76 | 3.86 | 0.0000 |
| Visit T1 | -0.51 | 0.25 | 0.0363 |
| Visit T2 | -0.79 | 0.25 | 0.0018 |
| LVD | 3.68 | 0.70 | 0.0000 |
| Age [years] | 0.27 | 0.04 | 0.0000 |
| Male gender | -1.89 | 0.57 | 0.0009 |
| BMI [kg/m2] | -0.06 | 0.06 | 0.3326 |
| Visit T1 x LVD | -0.54 | 0.56 | 0.3327 |
| Visit T2 x LVD | -0.08 | 0.59 | 0.8904 |
|  |  |  |  |
| *Peak VO_2_ [ml/kg/min]* |  |  |  |
| Intercept | 45.30 | 2.17 | 0.0000 |
| Visit T1 | 1.49 | 0.11 | 0.0000 |
| Visit T2 | 1.43 | 0.11 | 0.0000 |
| LVD | -2.15 | 0.39 | 0.0000 |
| Age [years] | -0.26 | 0.02 | 0.0000 |
| Male gender | 4.07 | 0.32 | 0.0000 |
| BMI [kg/m2] | -0.43 | 0.03 | 0.0000 |
| Visit T1 x LVD | 0.11 | 0.25 | 0.6422 |
| Visit T2 x LVD | 0.03 | 0.26 | 0.9083 |
|  |  |  |  |
| *SF36 Physical Component Score* |  |  |  |
| Intercept | 69.21 | 3.68 | 0.0000 |
| Visit T1 | 2.37 | 0.26 | 0.0000 |
| Visit T2 | 2.87 | 0.27 | 0.0000 |
| LVD | -2.43 | 0.70 | 0.0005 |
| Age [years] | -0.24 | 0.04 | 0.0000 |
| Male gender | 3.08 | 0.55 | 0.0000 |
| BMI [kg/m2] | -0.29 | 0.05 | 0.0000 |
| Visit T1 x LVD | 0.55 | 0.61 | 0.3680 |
| Visit T2 x LVD | 0.88 | 0.62 | 0.1580 |

LVD, left ventricular dysfunction; VE, ventilation; VCO2, carbon dioxide production; T1, CR start; T2, CR end; BMI, body mass index; VO2, oxygen consumption; SF36, Short-form quality of life questionnaire
